# SGOL2 is a novel prognostic marker and fosters disease progression via a MAD2-mediated pathway in hepatocellular carcinoma

**DOI:** 10.1186/s40364-022-00422-z

**Published:** 2022-11-15

**Authors:** Qingqing Hu, Qiuhong Liu, Yalei Zhao, Lingjian Zhang, Lanjuan Li

**Affiliations:** State Key Laboratory for Diagnosis and Treatment of Infectious Diseases, National Clinical Research Center for Infectious Diseases, Collaborative Innovation Center for Diagnosis and Treatment of Infectious Diseases, The First Affiliated Hospital, College of Medicine, Zhejiang University, Hangzhou, 310003 China

**Keywords:** SGOL2, MAD2, Cell cycle, Hepatocellular carcinoma, Prognosis

## Abstract

**Background:**

Shugoshin-like protein 2 (SGOL2) is a centromeric protein that ensures the correct and orderly process of mitosis by protecting and maintaining centripetal adhesions during meiosis and mitosis. Here, we examined the potential role of SGOL2 in cancers, especially in hepatocellular carcinoma (HCC).

**Methods:**

One hundred ninety-nine normal adjacent tissues and 202 HCC samples were collected in this study. Human HCC cells (SK-HEP-1 and HEP-3B) were employed in the present study. Immunohistochemistry, immunofluorescence, western blot, Co-Immunoprecipitation technique, and bioinformatic analysis were utilized to assess the role of SGOL2 in HCC development process.

**Results:**

Overexpression of SGOL2 predicted an unfavorable prognosis in HCC by The Cancer Genome Atlas database (TCGA), which were further validated in our two independent cohorts. Next, 47 differentially expressed genes positively related to both SGOL2 and MAD2 were identified to be associated with the cell cycle. Subsequently, we demonstrated that SGOL2 downregulation suppressed the malignant activities of HCC in vitro and in vivo. Further investigation showed that SGOL2 promoted tumor proliferation by regulating MAD2-induced cell-cycle dysregulation, which could be reversed by the MAD2 inhibitor M2I-1. Consistently, MAD2 upregulation reversed the knockdown effects of SGOL2-shRNA in HCC. Moreover, we demonstrated that SGOL2 regulated MAD2 expression level by forming a SGOL2-MAD2 complex, which led to cell cycle dysreuglation of HCC cells.

**Conclusion:**

SGOL2 acts as an oncogene in HCC cells by regulating MAD2 and then dysregulating the cell cycle, providing a potential therapeutic target in HCC.

**Supplementary Information:**

The online version contains supplementary material available at 10.1186/s40364-022-00422-z.

## Background

Hepatocellular carcinoma, a major type of primary liver cancer, is the third leading cause of cancer-related mortality globally [1]. Although recent improvements in the diagnosis and treatment of HCC are emerging, the prognosis of HCC patients and the treatment options for patients with advanced liver cancer are far from satisfactory when compared with those of other types of tumors [2, 3]. Hence, there is an urgent need to identify novel biomarkers for the early diagnosis and progression of HCC.

The precise separation of chromosomes is critical for the maintenance of genomic stability and function during mitosis [4, 5]. Genetic instability caused by chromosomal abnormalities may contribute to a variety of diseases, including cancers [6]. Shugoshins, including SGOL1 and SGOL2, were originally considered to be preservers of centromeric cohesion during meiosis and mitosis, which is fundamental for both chromatin structure and function [7, 8]. In the M phase of the cell cycle, shugoshins recruit PP2A to the centromere and act as a centromeric adaptor for protein phosphatase 2 A (PP2A) [9–12]. Shugoshin-like protein 2 (SGOL2) is a centromeric protein that associates with cohesin at centromeres and ensures the correct and orderly process of mitosis by protecting and maintaining centripetal adhesions during meiosis and mitosis [13, 14]. SGOL2 is also reportedly associated with chromatin condensation and the transcription of subtelomere genes [15]. As previously reported, SGOL2 specifically interacts with mitotic arrest deficient 2 like 1 (MAD2) and regulates the processes of cell mitosis [16], especially in the separation of eukaryotic sister chromatids. In general, SGOL2 can form a SGOL2–MAD2 complex upon binding with SAC-activated MAD2, which functions as a separase inhibitor [16]. In addition, activated MAD2 enables SGOL2 to bind and sequester separase during the cell cycle [16]. Therefore, the proper expression of SGOL2 is essential for maintaining normal physiological conditions, whereas the abnormal expression of SGOL2 can lead to the occurrence of disease. For example, R. Faridi reported that Perrault syndrome could be collectively caused by comutations of SGOL2 and CLDN14 [17]. Llano et al. demonstrated that mice with the depletion of SGOL2 survived normally without any obvious alterations but were sterile, indicating that SGOL2 plays a fundamental role in meiosis rather than in mitotic cell division in mice [18]. In addition, SGOL2 plays a critical role in tumorigenesis. A study from Canada demonstrated that the expression of SGOL2 was significantly different in patients with Sézary syndrome compared to healthy controls [19]. Moreover, SGOL1 expression was demonstrated to be upregulated in HCC and was associated with the early development of HCC, indicating that SGOL1 is a promising target [20]. However, the function of SGOL2 in HCC is unclear. Thus, further research on SGOL2 is urgently needed.

This study aimed to explore the biological function of SGOL2 in HCC through bioinformatics analysis and to clarify its probable mechanisms. We evaluated its expression profile by bioinformatics, which was further verified in 2 independent HCC cohorts. We also found that SGOL2 promoted HCC progression in vitro and in vivo. Further investigation demonstrated that SGOL2 can promote the expression of MAD2 by forming a SGOL2-MAD2 complex, which subsequently induces cell cycle dysregulation in HCC cells. Thus, the results of this study further our knowledge of SGOL2 and highlight its potential as a new therapeutic target for hepatocellular carcinoma.

## Methods

### Clinical specimens

This study was approved by the Research Ethics Committee of Zhejiang University. All experiments were performed according to regulations. A total of 199 normal adjacent tissues and 202 HCC samples were collected in this study. Part of the samples was obtained from Shanghai Outdo Biotech Company (Shanghai, China) (cohort 1, 97 pairs of matched HCC and normal adjacent tissues and 3 single HCC tissues). The other part was from the Department of Hepatobiliary Surgery, the First Affiliated Hospital of Zhejiang University (Zhejiang, China) (cohort 2, 102 pairs of matched HCC and normal adjacent tissues). The clinicopathological characteristics of all patients are shown in Table 1 (cohort 1), Tables 2 and 3 (cohort 1 + 2). None of HCC patients received any pre-surgery treatments, such as radiofrequency ablation (RFA), transcatheter arterial chemoembolization (TACE), immunotherapy and targeted therapy.

**Table 1:** Correlation between SGOL2 expression and clinicopathological characteristics in Cohort 1

Variables	SGOL2 expression		Total	χ2	*p*-value
	Low	High			
Age (year)					
≤ 58	25	23	48	1.733	0.188
> 58	19	30	49		
Null					
Sex					
Female	6	8	14	0.041	0.839
Male	38	45	83		
***Grade***					
1/2	40	33	73	10.236	***0.001***
3	4	20	24		
***T stage***					
T1/T2	33	29	62	4.289	***0.038***
T3/T4	11	24	35		
***TNM stage***					
Ι/II	33	29	62	4.289	***0.038***
III/IV	11	24	35		
***Thrombus***					
Negative	29	33	62	4.381	***0.036***
Positive	5	18	23		
Null			12		
Cirrhosis					
Negative	9	11	20	0.002	0.965
Positive	32	40	72		
Null			5		
HBsAg					
Negative	2	9	11		0.103*
Positive	39	42	81		
Null			5		
HBcAb					
Negative	0	2	2		0.496*
Positive	38	41	79		
Null			16		
TB					
Negative	23	36	59	0.531	0.466
Positive	15	17	32		
Null			6		
AFP					
Negative	23	35	58	0.356	0.551
Positive	13	15	28		
Null			11		
ALB					
Low	16	19	35	1.082	0.298
Normal/high	18	34	52		
Null			10		
GGT					
Negative	3	5	8		0.693*
Positive	15	14	29		
Null			60		
ALT					
Negative	16	30	46	2.859	0.091
Positive	25	23	48		
Null			3		
CD34					
Negative	1	1	2		1.000*
Positive	26	36	62		
Null			33		
***CK19***					
Negative	38	29	67	11.271	***0.001***
Positive	6	24	30		
Null					

*Fisher Test

**Table 2:** Differential expression of SGOL2 in liver cancer and adjacent tissues

Cohort 1	n	SGOL2 expression		Chi-square Value	*p*-value
		Low	High		
Liver cancer	97	44	53	45.336	< 0.001
Adjacent tissues	97	88	9		
Cohort 2	n	SGOL2 expression		Chi-square Value	*p*-value
		Low	High		
Liver cancer	102	21	81	88.436	< 0.001
Adjacent tissues	102	88	14

**Table 3:** Correlation between SGOL2 expression and clinicopathological characteristics in Cohort 1/2

	Cohort 1 (***n*** = 100)	Cohort 2 (***n*** = 102)
**Characteristics**	**No. of patients (%)**	**No. of patients (%)**
**Gender**		
Male	86 (86.0%)	81 (79.41%)
Female	14 (14.0%)	21 (20.59%)
**Age**		
≤ 58	50 (50.0%)	45 (44.12%)
> 58	50 (50.0%)	57 (55.88%)
**Liver cirrhosis history**		
Yes	21 (21.0%)	65 (63.73%)
No	73 (73.0%)	37 (36.27%)
Null	6 (6.0%)	
**TNM stages**		
I/II	63 (63.3%)	57 (55.88%)
III/IV	37 (37.0%)	45 (44.12%)

### Expression analysis

The mRNA level of SGOL2 in tumor versus normal tissues and liver cancer versus normal liver tissues was analyzed by Gene Expression Profiling Interactive Analysis (GEPIA) and Oncomine database. The protein level of SGOL2 in HCC was analyzed in the Human Protein Atlas (HPA) database and verified in cohort 2 by Western blot and cohort 1/2 by immunohistochemistry (IHC) staining. Next, we performed a subgroup analysis of SGOL2 mRNA expression using the liver hepatocellular carcinoma (LIHC) dataset from The Cancer Genome Atlas (TCGA) and the UALCAN database. The mRNA expression levels of SGOL2 were evaluated in various cell lines, including seventeen liver cell lines, in the CCLE database.

### Survival analysis

We performed Kaplan–Meier analysis for overall survival (OS), relapse-free survival (RFS), progression-free survival (PFS), and disease-specific survival (DSS) in the Kaplan–Meier Plotter database. In addition, patients in cohort 1 were separated into low-expression and high-expression groups based on the median expression value of SGOL2. Then, we performed overall survival analysis and compared survival curves by the log-rank test. Moreover, multivariate Cox proportional hazards regression analyses were performed to test whether SGOL2 was an independent prognostic factor in R software 3.6.0.

### Bioinformatic analysis

Please refer to the supplementary methods section.

### Cell lines, transfection, and reagents

The HCC cell lines SK-HEP-1 and HEP3B were purchased from Procell (Wuhan, China). All cells were cultured in DMEM (Gibco) supplemented with 10% fetal bovine serum (Gibco), 1% streptomycin, and penicillin. Cells were transfected with lentivirus or plasmid purchased from Shanghai Genomeditech (Shanghai, China) and verified by DNA sequencing. Lentiviruses containing shNC (negative control, NC) and shSGOL2 were constructed using the vector pGMLV-SC5. The shRNA sequence used to target SGOL2 was as follows: 5′-GGTCAGAATTCCCTAACTTGT-3′. The pGMLV-SGOL2 plasmid contained the SGOL2 coding sequence, and the pGMLV-MAD2 plasmid contained the MAD2 coding sequence. For plasmid transfection, SK-HEP-1 and HEP3B cells were seeded in 12-well plates and then transfected with plasmids (4 mg per well) using Lipofectamine 2000 Reagent (Invitrogen) according to the protocols. Cells were harvested for analysis after 48 h.

### Cell viability

Here, cells were seeded in 96-well plates at 2000 cells/well and incubated. Cell proliferation was evaluated by Cell Counting Kit-8 assays (CCK-8, APExBIO, USA) according to the protocol.

### Apoptosis and cell cycle analysis

For cell cycle analyses, cells were fixed with 70% ethanol at 4 °C overnight and stained with RNase A containing propidium iodide (Sigma–Aldrich, USA). Cell cycle distribution was determined using flow cytometry. For apoptosis analysis, cells were stained with an Annexin V-FITC/PI kit (BD Biosciences) and analyzed in a FACSAria II flow cytometer (BD Biosciences, San Jose, USA).

### Tumor sphere assay, colony formation assay, and Transwell migration and invasion assays

The tumor sphere assay, assay, colony formation and migration, and invasion assays of HCC cells were performed as previously described [21].

### Western blot analysis and coimmunoprecipitation (WB/COIP)

Western blots were performed as described previously [22]. CoIP was conducted as described previously using an IP/COIP kit (Absin, Shanghai, China) [23]. The following antibodies were used: SGOL2 antibody (Bethyl, A301–262A, for WB/Co-IP), MAD2 antibody (Bethyl, A300-301A, for WB/Co-IP), SGOL1 (Immunoway, YT4275), cyclin D1 antibody (CST, 55506), cyclin E1 antibody (CST, 20808), PCNA antibody (CST, 13110), normal rabbit IgG (CST, 2729), E-cadherin antibody (CST, 14472), N-cadherin antibody (CST, 13116), vimentin antibody (CST, 5741), MMP9 antibody (CST,13667), β-catenin antibody (CST, 8480), and fibronectin antibody (Abcam, ab268021), secondary antibody (CST,7074/7076), GAPDH antibody (CST, 5174).

### Quantitative real-time PCR

We conducted immunohistochemistry as previously described [22]. For qRT–PCR, the following primers were used:

human SGOL2, 5′-TAAAGCACAACAACAGGGCAT-3′ (forward) and.

5′-AGGCGAAGAAATGTGTTCTCAAA-3′ (reverse);

human MAD2, 5′-GGACTCACCTTGCTTGTAACTAC-3′ (forward) and.

5′-GATCACTGAACGGATTTCATCCT-3′ (reverse);

human SGOL1, 5′-AACTCAGCAGTCACCTCATCT-3′ (forward) and.

5′- TGCACCTACGTTTAGGCAGAG-3′ (reverse);

### Immunohistochemistry

We conducted immunohistochemistry as previously described [22]. For detection of apoptosis, samples were treated with a TUNEL BrightGreen Apoptosis Detection Kit (Vazyme, A112–03) according to the manufacturer’s instructions.

### Immunofluorescence assay

Cells were seeded on coverslips. The cells were fixed, permeabilized with 0.1% Triton X-100 for 1 min, blocked with 1% bovine serum albumin for 1 h, and treated with primary antibodies [SGOL2 antibody (Abcam, ab122258) and MAD2 antibody (Santa Cruz, C-10)] at 4 °C overnight. The cells were then treated with secondary antibodies and incubated for 1 h and DAPI for 10 min at room temperature. The images were generated from Confocal microscopy (Leica, Germany).

### Xenograft tumor models

Male BALB/c nude mice at 6 weeks of age were purchased from the Zhejiang Academy of Medical Sciences (Hangzhou, Zhejiang). Three mice were subcutaneously inoculated with SK-HEP-1 shNC or SK-HEP-1 shSGOL2 cells (1 × 10^7^ cells/200 μl serum-free DMEM). For the lung metastasis model, 7 mice were intravenously injected with SK-HEP-1 shNC or SK-HEP-1 shSGOL2 cells (3 × 10^6^ cells/200 μl serum-free DMEM) through the tail vein. The lung and liver metastatic nodules of HCC were analyzed by HE staining. Tumor volume was calculated by the following formula: tumor size = ab^2^/2. Three weeks after inoculation, animals were euthanized, and tumors were collected and fixed for immunohistochemical analysis. This animal study was approved by the Research Ethics Committee of Zhejiang University. All experiments were performed according to regulations.

### Statistical analyses

One-way ANOVA and two-tailed Student’s t-tests were employed to analyze the data. All data are presented as the mean ± standard deviation (SD). We described statistical significance as follows: NS, not significant; *P ≤ 0·05; **P ≤ 0·01; ***P ≤ 0·001; ****P ≤ 0·0001. We used GraphPad Prism software version 7.0 (GraphPad Software, San Diego, CA, USA) and SPSS 20.0 software (SPSS, Chicago, IL, USA) to perform the statistical analysis.

## Results

### High expression of SGOL2 in HCC

**Fig. 1:**
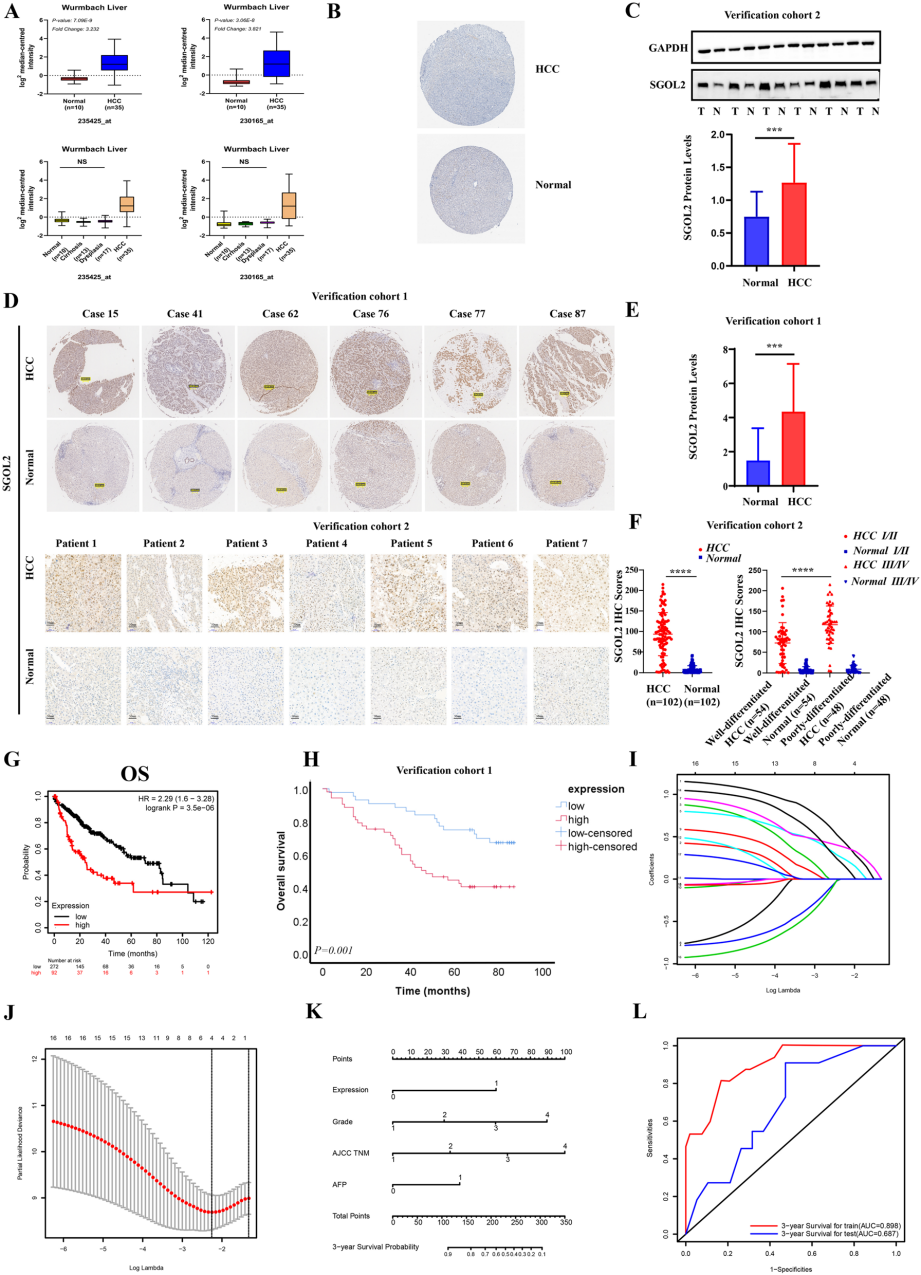
High expression of SGOL2 in HCC. **A** SGOL2 was overexpressed in HCC in Wurmbach liver database. **B** Protein expression of SGOL2 was elevated in HCC patients in HPA database. **C** Immunoblot analysis of SGOL2 in HCC samples and paracancerous tissues from patients in cohort 2, and GAPDH was used as a loading control. **D**-**F** SGOL2 staining of paired clinical specimens, and the statistic quantification results in cohorts 1 and 2(*n* = 202). Protein expression of SGOL2 in the poorly differentiated group was significantly higher than that in the well-differentiated group of cohort 2. **G** TCGA dataset analysis of the relationship between the SGOL2 expression levels and the prognosis of HCC patients (*n* = 364). **H** Overall survival (OS) analysis of HCC patients with high SGOL2 expression or low SGOL2 expression in cohort 1 (*n* = 100). **I**-**J** Identification of the optimal penalization coefficient lambda (λ) in the Lasso model in cohort 1. **K** The nomogram based on SGOL2 for predicting the prognosis of HCC patients in cohort 1. **L** ROC curve analysis was used to evaluate the performance of this nomogram for 3-year overall survival prediction in the training and validation groups. OS, overall survival; RFS, relapse-free survival; PFS, progression-free survival; DSS, disease-specific survival; HR, hazard ratio. The results are presented as the mean ± SD, *P < 0.05, **P < 0.01, ***P < 0.001, ****P < 0.0001

We found that SGOL2 mRNA expression was upregulated in different tumors, including hepatocellular carcinoma, colorectal cancer, and breast cancer, in the Oncomine database (Fig. S1A). In addition, we further searched the GEPIA database to systematically assess the expression profile of SGOL2 in a variety of carcinomas (Fig. S1B). To better understand its expression levels in different diseases, including HCC, cirrhosis, and dysplasia, we selected and analyzed data from the Wurmbach liver dataset (Fig. 1A). The results showed that SGOL2 expression was dramatically upregulated in HCC tissues compared with normal tissues (*P* < 0.05), whereas there were no significant differences among the cirrhosis, dysplasia, and normal liver tissue groups. Moreover, SGOL2 protein levels were analyzed using the HPA database. As shown in Fig. 1B, SGOL2 was weakly expressed in the tumor tissue (Patient ID: 3196) and negatively expressed in normal liver tissue (Patient IDs: 2429). Furthermore, we analyzed the protein levels of SGOL2 in HCC and matched adjacent non-tumor tissues in cohort 2 by Western blots and found that SGOL2 expression was extremely upregulated in HCC (Fig. 1C), and immunohistochemistry (IHC) staining from the two independent HCC cohorts confirmed the results (Fig. 1D-F). In addition, we divided the patients in cohort 2 into 2 groups according to differentiation levels: a well-differentiated group and a poorly differentiated group. Interestingly, the expression levels of SGOL2 in the less differentiated group were markedly higher than those in the well-differentiated group, indicating that the expression level of SGOL2 is directly proportional to tumor progression (Fig. 1F). Similarly, we found the same result: SGOL2 expression in the Grade 3 group was dramatically higher than that in the Grade 1/2 group in cohort 1 (Table 1, *p* = 0.001).

To expand the number of patients included in the analysis, we confirmed the overexpression of SGOL2 in the TCGA-HCC database. We found that the expression level of SGOL2 showed a positive association with grade levels (Fig. S3G), consistent with our previous results in cohorts 1 and 2. Moreover, SGOL2 was found to be roughly proportional to the stage levels (Fig. S3B). The mRNA level of SGOL2 was higher in the HCC than in the non-tumor tissues (Number of T vs *N* = 371 vs 50) (Fig. S3A). We also conducted subgroup analysis in various subgroups (race, sex, age, weight), which showed significantly elevated SGOL2 expression levels (Fig. S3C-F). Furthermore, SGOL2 expression was significantly elevated in TP53-mutant patients (Fig. S3I). Interestingly, no difference was shown between the HCC patients with and without lymph node metastasis (Fig. S3H). Thus, these results indicated that SGOL2 overexpression was related to the development of HCC.

### Upregulation of SGOL2 expression indicated poor prognosis in HCC patients

To determine whether SGOL2 could be a novel prognostic marker in HCC, we analyzed its prognostic significance in HCC patients. SGOL2 overexpression was closely related to poor overall survival (HR = 2.29 (1.6–3.28), *P* = 3.5e-06), relapse-free survival (HR = 1.96 (1.38–2.78), *P* = 0.00013), progression-free survival (HR = 2.1 (1.55–2.84), *P* = 9.2e-07) and disease-specific survival (HR = 2.84 (1.81–4.47), *P* = 2.3e-06) in HCC patients (Fig. 1G, Fig. S4A-C). To better identify the negative relationship between SGOL2 expression and the prognosis of hepatocellular carcinoma, we performed survival analysis in verification cohort 1. According to the immunohistochemical score, we divided the patients (*n* = 97) in cohort 1 into a low expression group (*n* = 44) and a high expression group (*n* = 53), and then performed survival analysis. As shown in Fig. 1H, we found that the overall survival of the low expression group was markedly higher than that of the high expression group (*p* = 0.001). Subsequently, we conducted a SGOL2-based prognostic model. All HCC patients in cohort 1 were randomly divided into two groups: the training group (*n* = 67) and the validation group (*n* = 30). Next, we identified four variables that were closely related to survival: expression, grade, AJCC TNM, and AFP based on the lasso regression model (Fig. 1I-J). Based on the multivariable Cox proportional hazards model, we also predicted the 3-year survival of HCC patients using a nomogram (Fig. 1K). We also assessed the discrimination power of this nomogram by receiver operating characteristic (ROCs) curves. The area under the ROC curve for the 3-year survival probability of the training group and the validation group were 0.898 and 0.687, respectively (Fig. 1L, Fig. S4D-E). The calibration curves of the nomogram showed good probability consistencies between the two groups (Fig. S4F-G). In conclusion, high SGOL2 expression indicated a poor prognosis in HCC patients.

### SGOL2 promoted HCC cells growth, stemness, migration and invasion

**Fig. 2:**
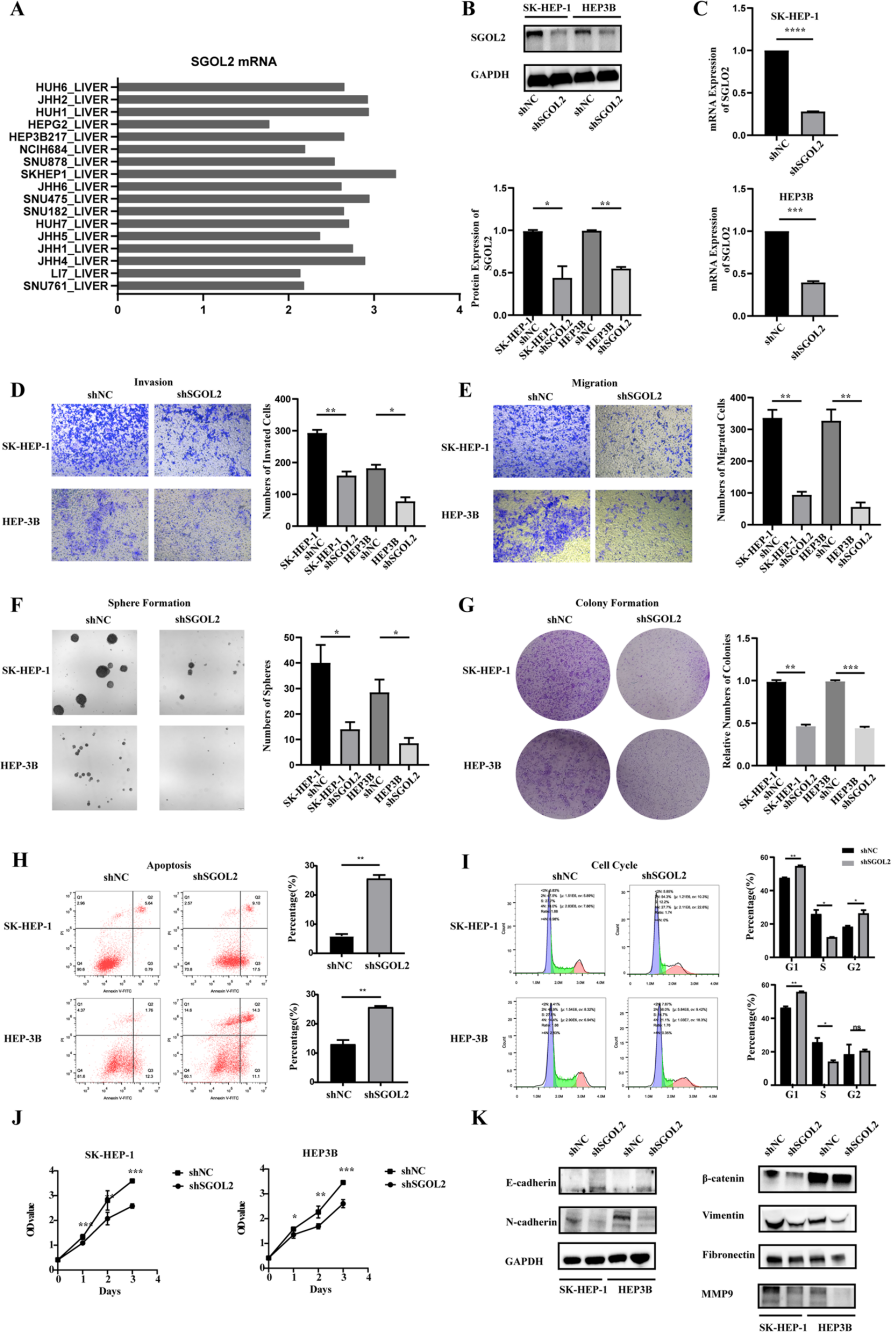
Downregulation of SGOL2 expression inhibited the malignant behaviors of HCC cells in vitro. **A** The mRNA level of SGOL2 in liver and HCC cell lines. **B**-**C**, SK-HEP-1, and HEP3B cells were transfected with shNC or shSGOL2 lentivirus, and the knockdown of SGOL2 at the mRNA and protein levels was validated by RT–PCR and Western blots, respectively. GAPDH was used as a loading control. **D**-**G** Invasion, migration, sphere formation, and colony formation assays of the SGOL2-downregulated HCC cells were detected and analyzed. **H**-**I** Downregulation of SGOL2 expression induced cell cycle arrest in the G1/S phase and activated the apoptosis of HCC cells. **J** SK-HEP-1, and HEP3B cells were transfected with shNC or shSGOL2, and the proliferation of HCC cells was detected at Days 0, 1, 2, and 3 by CCK-8 assays. **K** Effect of SGOL2 on the EMT in HCC cell lines. SK-HEP-1 and HEP3B cells were transfected with shNC or shSGOL2, and Western blots were used to detect the levels of E-cadherin, N-cadherin, β-catenin, Vimentin, fibronectin, and MMP9. The results are presented as the mean ± SD, *P < 0.05, **P < 0.01, ***P < 0.001, ****P < 0.0001

SK-HEP-1 and HEP3B cells were chosen to evaluate the function of SGOL2 according to the mRNA levels of SGOL2 in different HCC cell lines based on the data from CCLE (Fig. 2A). After transfection with lentivirus, we confirmed that SGOL2 was significantly decreased at both the mRNA and protein levels (Fig. 2B-C). Transwell assays indicated that low SGOL2 expression suppressed the migration and invasion of HCC cells (Fig. 2D-E). Moreover, we tested the expression alterations of key EMT-related proteins responding to the downregulation of SGOL2 expression (Fig. 2K). Interestingly, the results showed that shSGOL2 resulted in increased expression of E-cadherin and reduced expression of N-cadherin, fibronectin, vimentin, β-catenin, and MMP9. Thus, downregulation of SGOL2 expression could inhibit cell metastasis by repressing migration, invasion, and EMT in HCC. Next, a sphere formation assay was performed to determine whether stemness could be influenced by downregulating SGOL2 expression. Consistently, the spheres in the shSGOL2 group were dramatically fewer and smaller than those in the shNC group (Fig. 2F). Furthermore, the shSGOL2 group developed fewer cell colonies than the NC group (Fig. 2G), and we also observed that low SGOL2 expression suppressed the proliferation of HCC cells by CCK-8 assays (Fig. 2J). In addition, flow cytometry-based assays demonstrated that apoptotic indices in the shSGOL2 group were dramatically higher than those in the NC group (Fig. 2H), and the cell cycle was strongly influenced by the downregulation of SGOL2 expression (Fig. 2I).

### SGOL2 dysregulated the cell cycle process by regulating the MAD2 protein

**Fig. 3:**
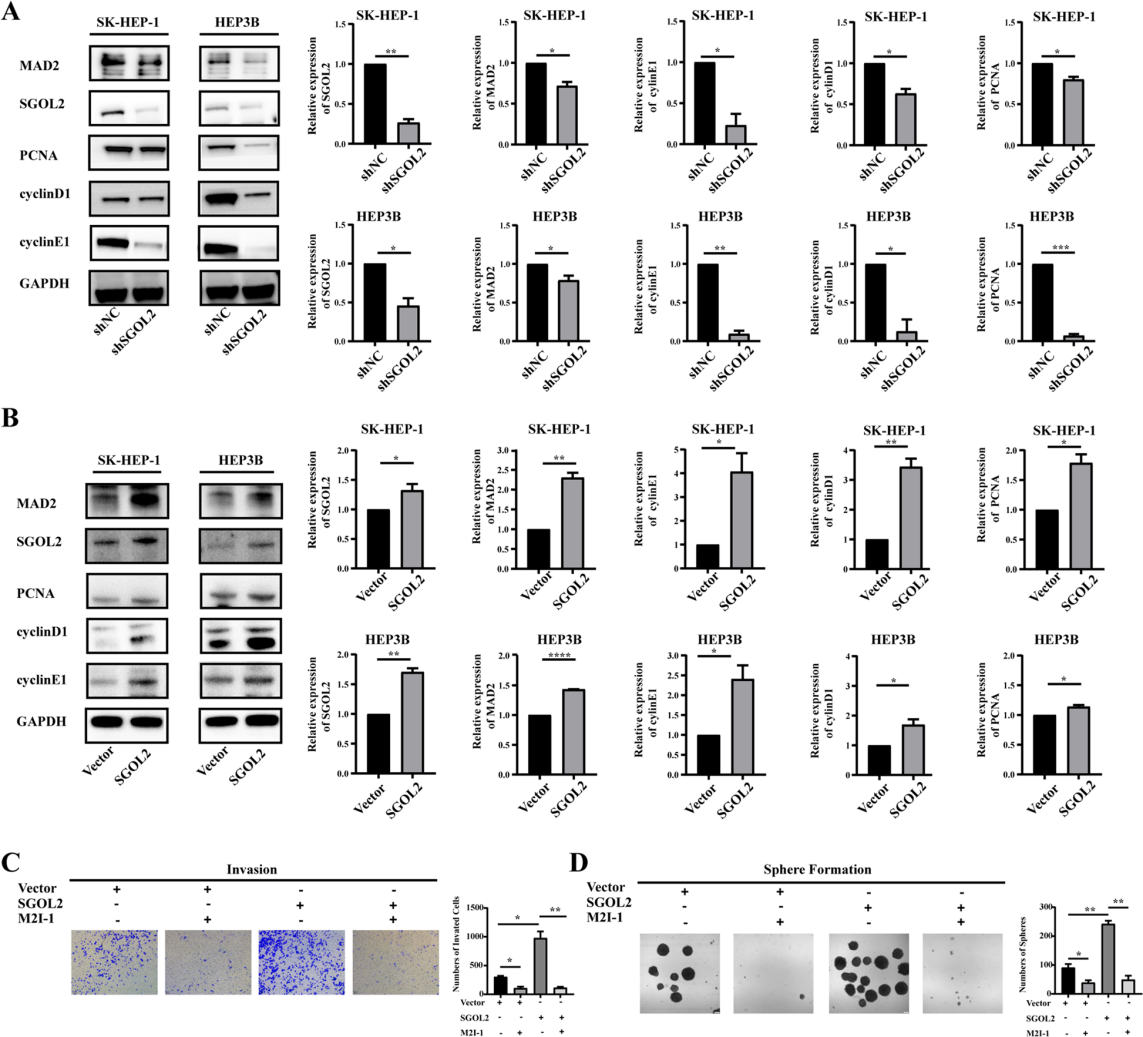
SGOL2 dysregulated the cell cycle by regulating MAD2 in HCC cells. **A** SK-HEP-1 and HEP3B cells were transfected with shNC or shSGOL2, and the levels of PCNA, cyclin D1, cyclin E1, SGOL2, and MAD2 were detected by Western blots to study the effect of SGOL2 on cell cycle and MAD2. GAPDH was used as a loading control. **B** SK-HEP-1, and HEP3B cells were transfected with SGOL2 plasmid or vector control plasmid, and the levels of PCNA, cyclin D1, cyclin E1, SGOL2, and MAD2 were detected by Western blots. GAPDH was used as a loading control. **C**-**D**, Invasion and sphere formation of SGOL2-upregulated HCC cells with or without M2I-1 treatment were detected and analyzed. The results are presented as the mean ± SD, *P < 0.05, **P < 0.01, ***P < 0.001, ****P < 0.0001

**Fig. 4:**
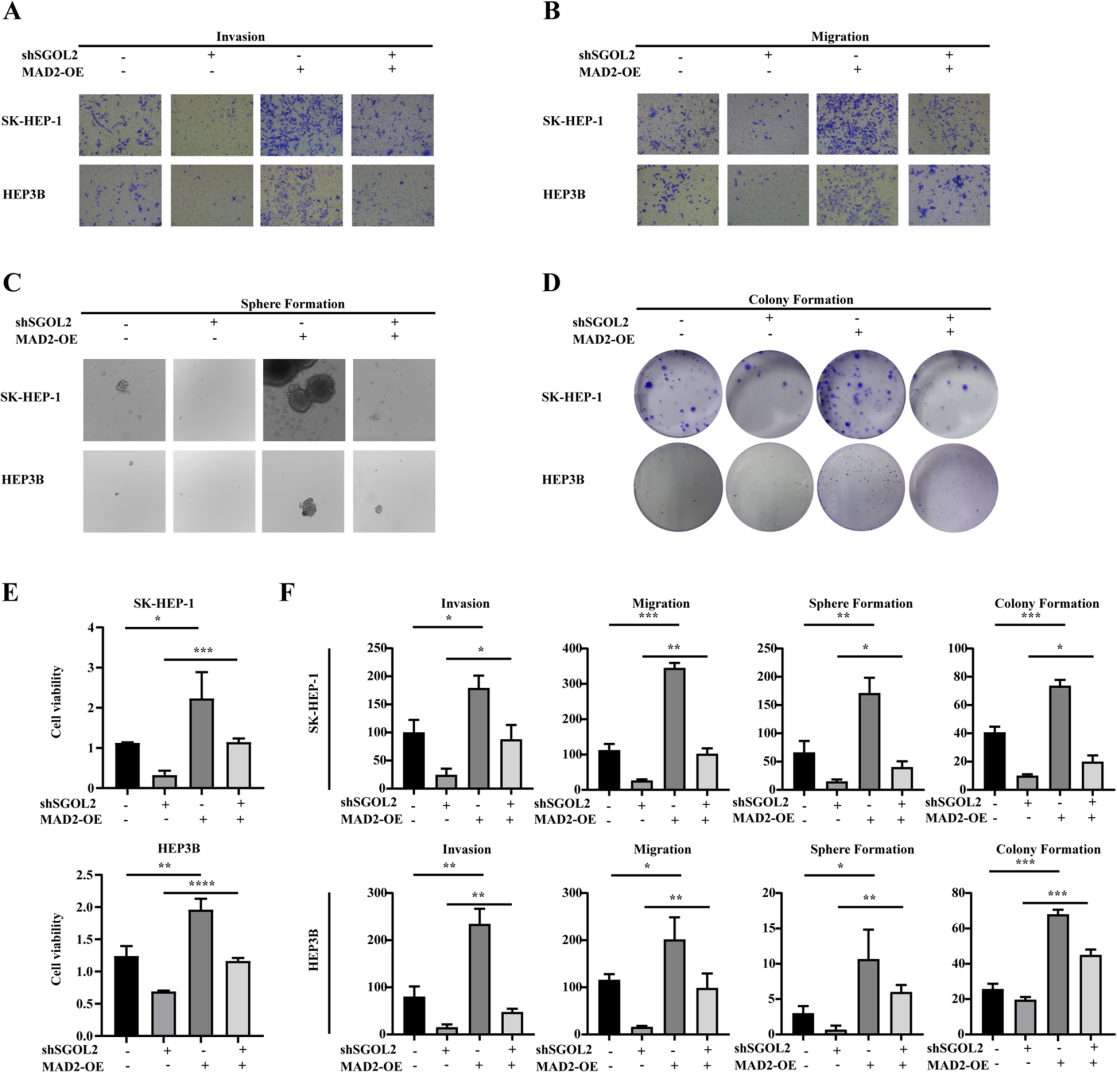
Overexpression of MAD2 reversed the knockdown effects of SGOL2-shRNA in HCC. **A**-**D**, **F** Invasion, migration, sphere formation, and colony formation assays of SGOL2 knockdown HCC cells with or without MAD2 overexpression were performed. **E** SK-HEP-1, and HEP3B cells were transfected with shSGOL2 or MAD2 plasmid, and the proliferation of HCC cells was detected at Days 3 by CCK-8 assays. The results are presented as the mean ± SD, *P < 0.05, **P < 0.01, ***P < 0.001, ****P < 0.0001

To validate these data through the bioinformatics analysis above, we further demonstrated the role of SGOL2 in HCC cells, especially in the cell cycle process, based on the above results. First, the protein level of MAD2 was highly declined in HCC cells through downregulation of SGOL2 expression, while overexpression of SGOL2 increased the expression of MAD2 (Fig. 3A-B). After the knockdown of SGOL2, the protein levels of PCNA, cyclin D1, and cyclin E1 were significantly decreased (Fig. 3A), whereas upregulation of SGOL2 expression strongly increased the expression of PCNA, cyclin D1, and cyclin E1 (Fig. 3B). Furthermore, when MAD2 was blocked by its specific inhibitor M2I-1, highly aggressive malignant behaviors of HCC cells caused by overexpression of SGOL2 were significantly reversed (Fig. 3C-D). A rescue assay was performed to confirm that the knockdown effect of SGOL2 shRNA could be reversed by the overexpression of MAD2. As shown in Fig. 4A-B, the number of migrated or invaded cells in the lower chamber of the shSGOL2 + MAD2 group was much more than that of the shSGOL2 group. Moreover, the number of spheres or colonies in the shSGOL2 + MAD2 group rose sharply compared to that in the shSGOL2 group, as shown in Fig. 4C-D. As shown in Fig. 4E, CCK-8 assays demonstrated that the upregulation of MAD2 expression could reverse the inhibitory effect of SGOL2 knockdown on the viability of HCC cells. From the above results, we can conclude that overexpressed MAD2 could reverse the knockdown effect of SGOL2 shRNA in HCC. Altogether, these data indicated that SGOL2 dysregulated the cell cycle and promoted the development of HCC by regulating the MAD2 protein.

**Fig. 5:**
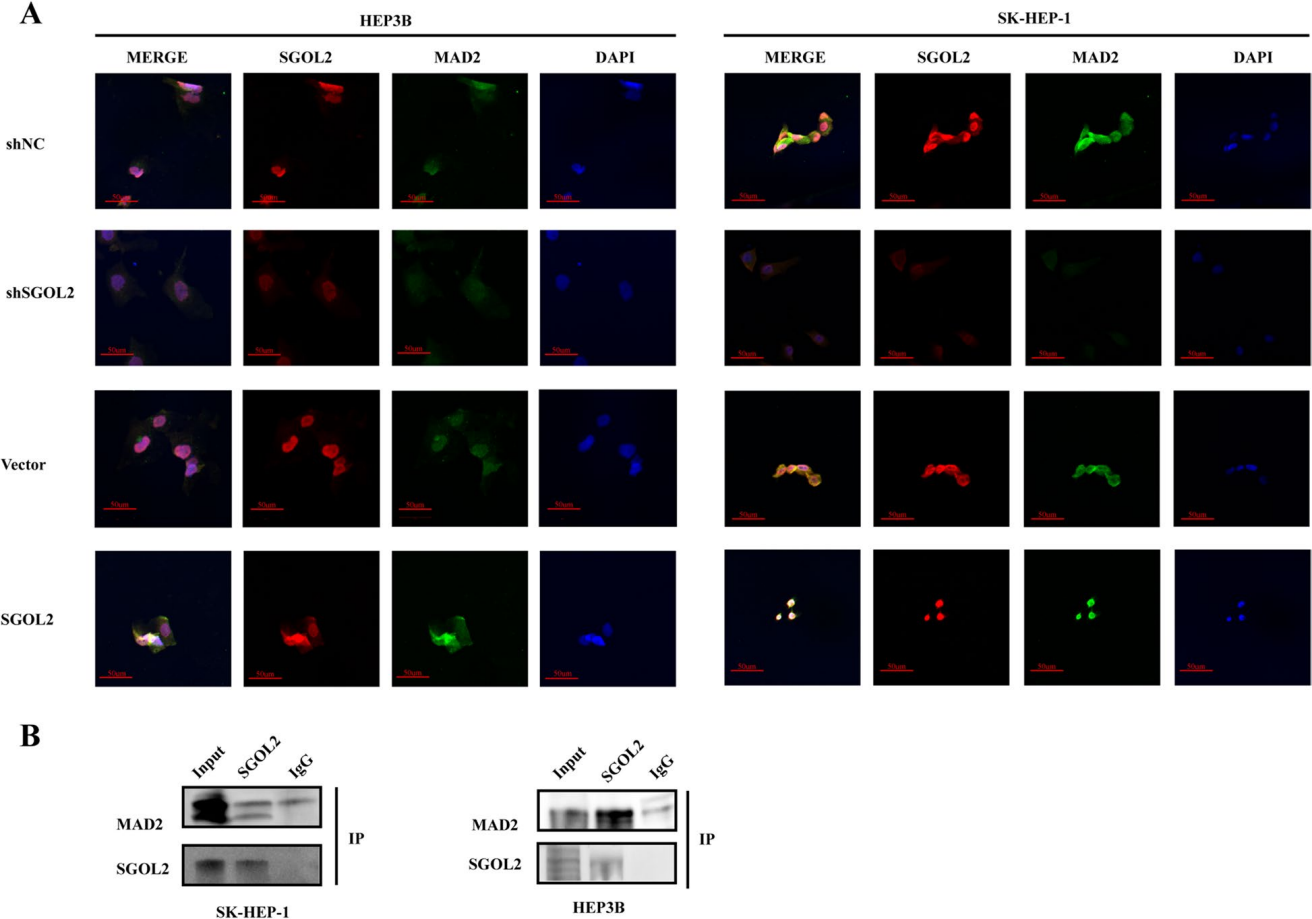
SGOL2 exerted its effect by forming a SGOL2-MAD2 complex. **A** SK-HEP-1 and HEP3B cells were transfected with shSGOL2 lentivirus or SGOL2 plasmid. The colocalization between SGOL2 (Red) and MAD2 (Green) was visualized as yellow fluorescence in the merged panel by Confocal microscopy. **B** The endogenous interaction between SGOL2 and MAD2 was detected by IP assays in HCC cells

Next, we examined how SGOL2 interacts with MAD2. Immunofluorescence (IF) staining showed that SGOL2 colocalized with MAD2 in both SK-HEP-1 and HEP3B cell lines by Confocal microscopy (Fig. 5A), and the coimmunoprecipitation (Co-IP) assay further verified that SGOL2 could bind with MAD2 (Fig. 5B). Altogether, these data collectively verified that SGOL2, binding with MAD2 and forming a SGOL2-MAD2 complex, regulated MAD2 and then fueled tumor cell growth by dysregulating the cell cycle process, which finally promoted the malignant behaviors of HCC cells, including proliferation, migration, invasion, stemness and EMT (Fig. 8J).

### SGOL2 knockdown inhibited HCC growth and metastasis in vivo

**Fig. 6:**
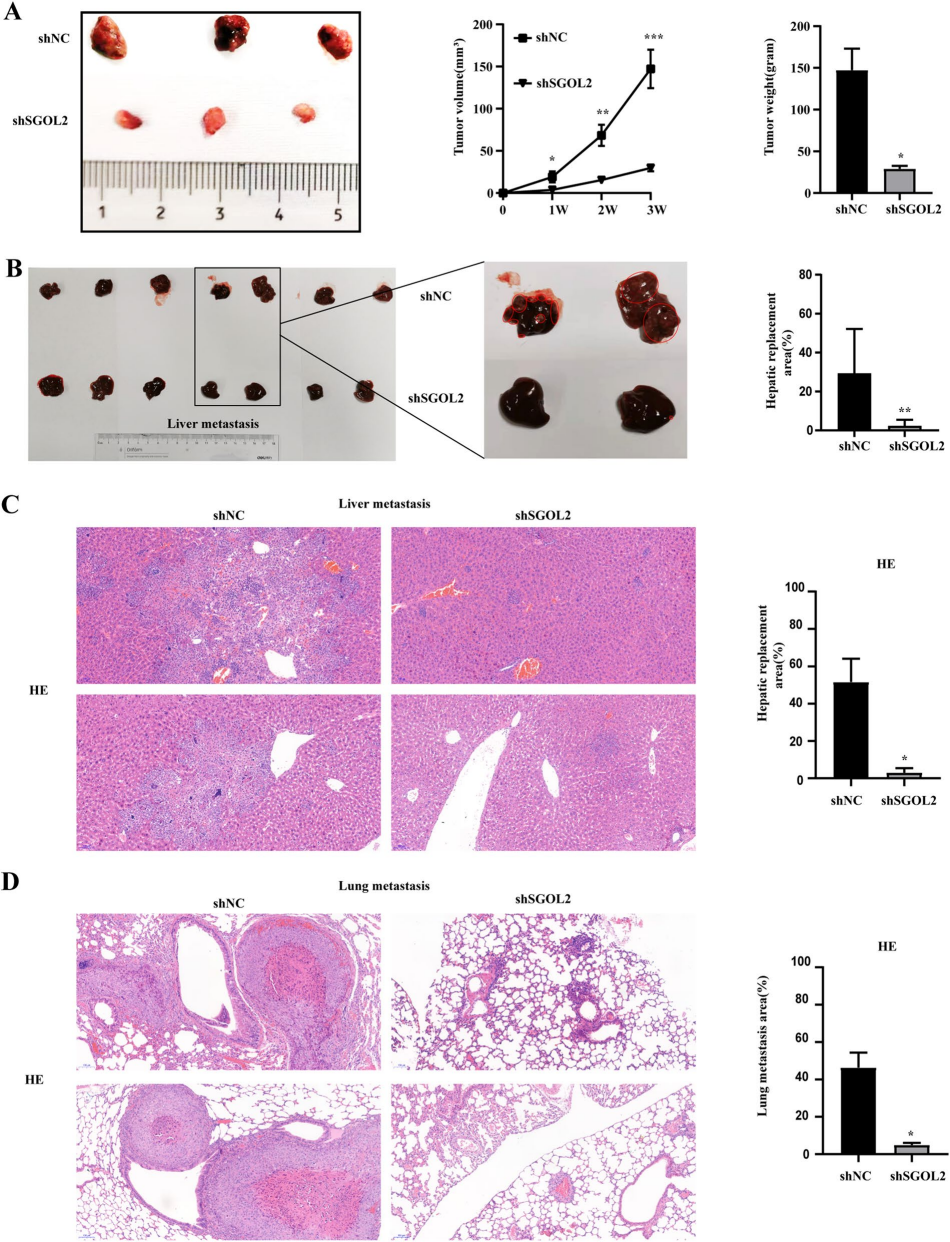
SGOL2 knockdown inhibited HCC growth and metastasis in vivo. **A**-**B** Xenograft model was set up to study the effects of SGOL2 on HCC tumor growth in vivo. Mice were divided into two groups and inoculated with SK-HEP-1 shNC or SK-HEP-1 shSGOL2 cells (s.c. *n* = 3, i.v. *n* = 7). Images of the isolated livers and tumors from sacrificed mice are presented, and the hepatic replacement area (HRA%) and the tumor volumes and tumor weights of the indicated groups were analyzed and compared. Loss of SGOL2 in SK-HEP-1 cells contributed to the reduction in tumorigenesis. **C**-**D**, HE staining of metastatic tumors in liver and lung tissues. Representative images and quantitative analysis results are shown

To further verify the role of SGOL2 in HCC in vivo, we constructed xenograft tumor models by SK-HEP-1 shNC and SK-HEP-1 shSGOL2. The mice were sacrificed on Day 21 after inoculation, and the formed tumors, lung, and liver were statistically analyzed (Fig. 6). Both the volumes and weights of the formed tumors were dramatically decreased in the shSGOL2 group compared with the shNC group (Fig. 6A). We also analyzed lung or liver metastatic area by HE in the metastatic tumors (Fig. 6C-D). Both the area of the metastatic lung or liver tumors was dramatically decreased in the shSGOL2 group compared with the shNC group (Fig. 6C-D).

**Fig. 7:**
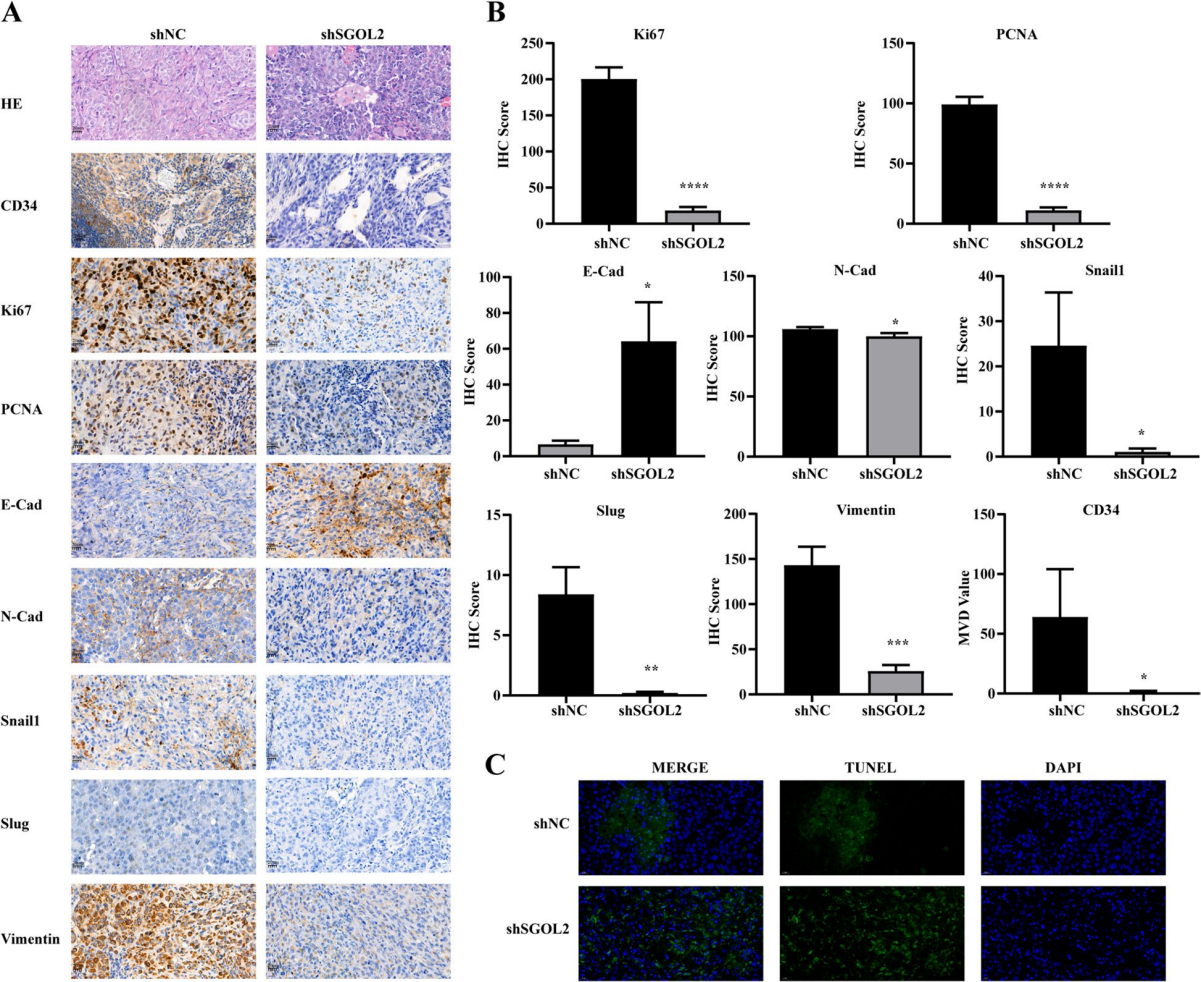
Downregulation of SGOL2 expression promoted apoptosis in vivo. **A**-**B** IHC staining of CD34, Ki-67, PCNA, and EMT-related markers. Representative images and quantitative analysis results are shown. **C** The apoptotic area was dramatically increased in the shSGOL2 group compared with the shNC group. The results are presented as the mean ± SD, *P < 0.05, **P < 0.01, ***P < 0.001, ****P < 0.0001

We further analyzed angiogenic markers (CD34), proliferative markers (Ki-67 and proliferating cell nuclear antigen [PCNA]), and EMT-related markers (E-cadherin, N-cadherin, vimentin, Snail, and Slug) by IHC in the formed tumors. Downregulation of SGOL2 expression resulted in the suppression of both proliferation and metastasis (Fig. 7A-B), which was consistent with the above in vitro results. We also found that the apoptotic area in the shSGOL2 group was much larger than that in the shNC group (Fig. 7C). Thus, these data indicated that SGOL2 promoted tumor growth and metastasis. To further validate our results, we also tested both the mRNA and protein levels of SGOL1 after the knockdown of SGOL2 in HCC cell lines by PCR and Western blots, respectively. As shown in Fig. S2 A-B, we found that the reduction in SGOL2 did not alter the expression of SGOL1. Thus, SGOL2 KD repressed the development of HCC by knocking down SGOL2 but not SGOL1.

### SGOL2 and MAD2 are associated with diverse signaling pathways and the prognosis of HCC patients

**Fig. 8:**
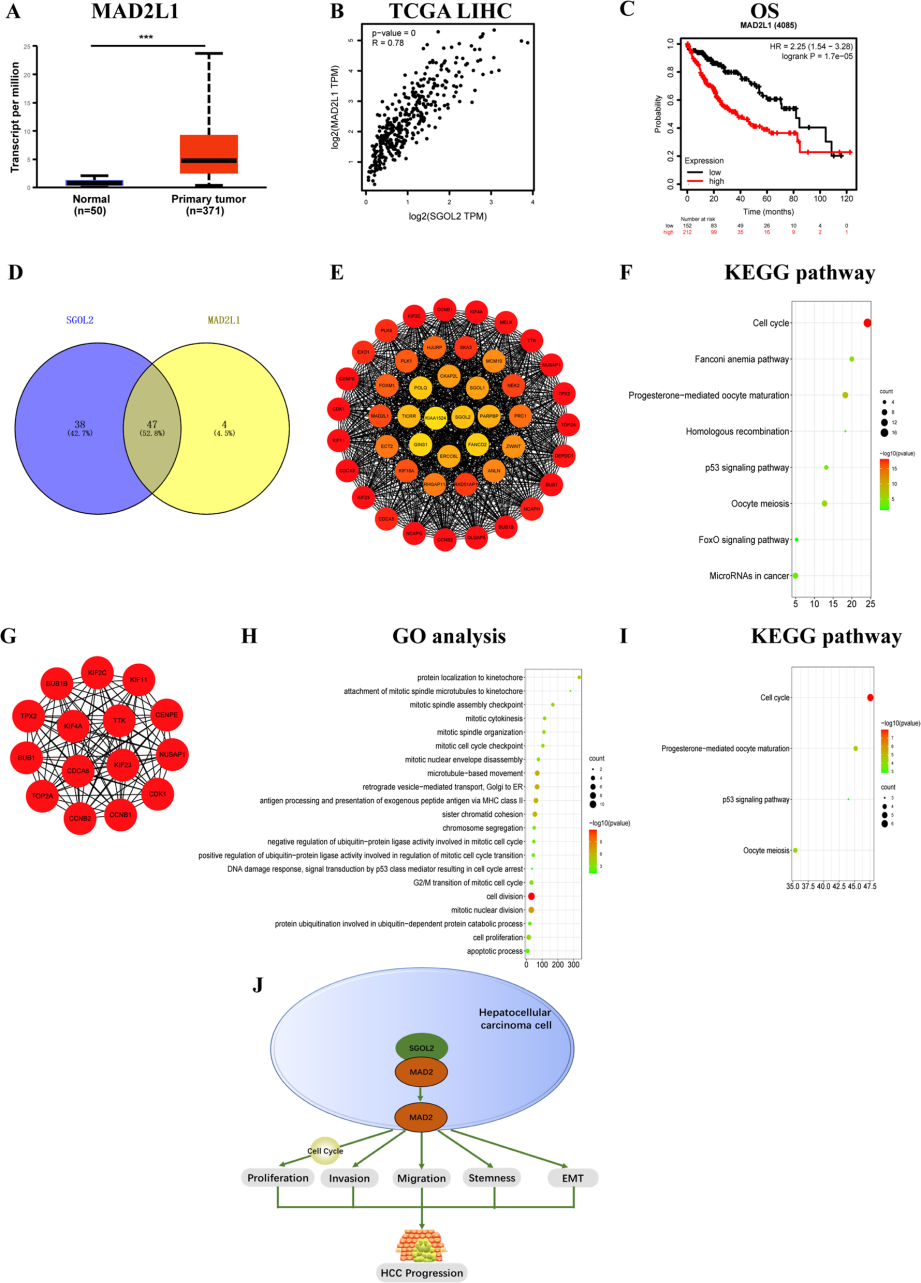
Upregulated MAD2 expression predicted poor prognosis in HCC and hub gene analysis positively related to both SGOL2 and MAD2. **A** MAD2 mRNA level is higher in HCC tissues than that in normal liver tissues (UALCAN). **B** MAD2 has a positive relation with SGOL2 in HCC (GEPIA). **C** Highly expressed MAD2 predicted poor prognosis in HCC. **D** Forty-seven genes positively correlated with both SGOL2 and MAD2, as shown by Venn diagram analysis. **E** The interaction network of the 47 genes. **F** KEGG enrichment of the 47 genes. **G** The graph shows the interaction network of the top 15 hub genes. **H**-**I** GO analysis and KEGG enrichment of the top 15 hub genes. HR: hazard ratio. **J** The role and mechanism of SGOL2 in HCC cells. SGOL2 forms a SGOL2-MAD2 complex and further regulates MAD2, resulting in dysregulation of the cell cycle and finally enhancing HCC malignant behaviors

Next, we tried to clarify the signal transduction pathway of SGOL2 in HCC cells. SGOL2 and MAD2 were reported to be involved in the separation of eukaryotic sister chromatids during the cell cycle [24]. Thus, we hypothesized that SGOL2 promotes tumors by influencing the expression of MAD2. To explore the role of MAD2, a factor closely related to SGOL2, in liver cancer, we used the UALCAN database to analyze its expression profile, clinical value, and prognostic significance. As shown in Fig. 8A, MAD2 expression was also markedly upregulated in HCC. Moreover, the expression of MAD2 in HCC showed a positive correlation with SGOL2 in the TCGA database (*R* = 0.78, *P* = 0) (Fig. 8B). Interestingly, we observed that high MAD2 expression was also related to unfavorable OS in HCC patients (Fig. 8C).

To discover SGOL2-MAD2 associated potential pathways in HCC patients, we used the data from LinkedOmics to identify differentially expressed genes related to both SGOL2 and MAD2 in HCC by Spearman’s test (Fig. S6A, D). The top 50 positively or negatively correlated markers were represented in Fig. S6B-F. Then, the positively correlated genes with coefficients > 0.8 were selected for further analysis. In total, we identified 85 genes positively associated with SGOL2 and 51 genes positively related to MAD2. Among these, 47 genes were positively related to both SGOL2 and MAD2 (Fig. 8D). Then, we constructed a PPI network based on the 47 differentially expressed genes using STRING and Cytoscape (Fig. 8E) and used it for KEGG enrichment analysis (Fig. 8F). The top 15 hub genes of the network were chosen for further analysis using cytoHubba based on the clusters identified in the PPI network using MCODE (Fig. 8G). Biological processes, such as cell division, cell proliferation, and apoptotic processes, were significantly affected and enriched based on GO analysis results (Fig. 8H). The co-expressed genes were mainly involved in the cell cycle, progesterone-mediated oocyte maturation, oocyte meiosis, and the p53 signaling pathway based on KEGG results (Fig. 8I). Then, we tried to assess whether these identified hub genes were related to prognosis. All 15 genes were significantly related to poor OS (BUB1B, NUSAP1, TTK, CCNB2, TOP2A, KIF2C, CCNB1, KIF23, TPX2, KIF11, KIF4A, CDK1, BUB1, CENPE, CDCA8) (Fig. S7).

## Discussion

The accurate separation of duplicated genomes in mitosis is fundamental for cells [25]. Chromosome segregation errors can lead to chromosomal instability (CIN), which induces tumorigenesis [26–31]. CIN could also lead to diversity in somatic copy number alterations (SCNAs), potentially providing the fundamental basis for tumor development and progression [25, 32–34]. SGOL2 is fundamental for separating sister chromatids [9]. A previous study revealed that SGOL2 together with MAD2 was closely related to the spindle assembly checkpoint (SAC) [16]. HCC is characterized by dysfunctional cell cycle progression and uncontrolled rapid proliferation [35–37]. However, it is not clear whether SGOL2 has any role in HCC and its function. Here, we analyzed the role of SGOL2 in HCC and demonstrated that its overexpression promoted the development and progression of HCC, while its deficiency suppressed tumorigenesis. We further validated that SGOL2 acted as an oncogene by forming a SGOL2-MAD2 complex and then dysregulated the cell cycle process in HCC.

MAD2 is a key protein in the spindle assembly checkpoint (SAC), encoded by an 11.5 kbp gene on chromosome 4q27 [38, 39]. In previous studies, MAD2 formed the MAD2-CDC20 complex and further combined with Mad3 (BubR1) and Bub3 to form the mitotic checkpoint complex that inhibits APC/C [40, 41]. Another report showed that closed MAD2 bound to MAD1, forming a MAD1/MAD2 complex, acting as a template for MAD2 bound to CDC20 in the spindle assembly checkpoint [38]. It has also been reported that MAD2 is overexpressed and correlated with cancer progression in different types of cancers, including colon, pancreatic, liver, and lung cancers [42]. Moreover, MAD2-overexpressing patients may benefit from MAD2-targeted therapy, which could dramatically dysregulate the cell cycle, effectively activate apoptosis and weaken the proliferation, metastasis, and stemness of tumor cells [43–47]. It was also reported that knockdown of MAD2 induced apoptotic signal transduction and increased the sensitivity of lung cancer cells to cisplatin [43]. Thus, targeting MAD2 is well recognized as an efficient cancer manipulation strategy. According to a previous report [24], SGOL2 combined with MAD2 is involved in the regulation of the cell cycle process. In addition, we found that the cell cycle may be regulated by SGOL2 and MAD2 based on protein-protein interaction (PPI) network analysis and pathway enrichment analysis. Therefore, we assumed that MAD2 may be the downstream target of SGOL2, and we further conducted loss-of-function and rescue tests. We found that loss of SGOL2 significantly suppressed the expression of MAD2 and markers related to the cell cycle. Furthermore, the promoting effect of upregulated SGOL2 expression on the malignant behaviors of HCC cells was dramatically reversed by the addition of the MAD2-specific inhibitor M2I-1. Consistently, the upregulation of MAD2 expression reversed the knockdown effects of SGOL2 shRNA in HCC. Therefore, we concluded that downregulation of SGOL2 expression inhibited the expression of MAD2, thereby reducing the levels of cyclin D1 and cyclin E1, inducing cell cycle arrest in the G1/S phase, and inhibiting the proliferation of HCC cells.

The regulation of MAD2 by SGOL2 has been investigated, and we are further interested in clarifying the molecular mechanism. As reported, SGOL2-MAD2 functions by manipulating the separation of eukaryotic sister chromatids during the cell cycle [24]. Intriguingly, our study showed that the expression of MAD2 was positively correlated to the expression of SGOL2. Immunofluorescence assays showed that SGOL2 colocalized with MAD2, which was further validated by a Co-IP assay, indicating the close link between SGOL2 and MAD2 and that the two factors may function together. In mice, the SGOL2 binds to MAD2 directly and MAD2 interaction Motif in the SGOL2 sequence is from 148 to 151. Michael Orth et al. found that MAD2 is a novel interaction partner of human SGOL2 but not SGOL1 [16]. In addition, it was demonstrated that SGOL2 could directly bind MAD2 and then functionally replace securin and sequesters most separase in securin-knockout cells [24]. Thus, we can conclude the direct binding of SGOL2 and MAD2, and more efforts should be made to identify the key regions for SGOL2-MAD2 interactions in the future. In this study, our data demonstrated that SGOL2, by forming a SGOL2-MAD2 complex, increased the expression of MAD2 and further promoted tumor growth in HCC. However, the evidence for the direct interaction between SGOL2 and MAD2 was quite a week. To confirm the direct binding of SGOL2 and MAD2, biochemical or biophysical assays such as Surface Plasmon Resonance (SPR), Isothermal Titration Calorimetry (ITC), Microscale Thermophoresis (MST), and Biolayer Interferometry (BLI) may better quantify biomolecular interactions.

There are fewer reports of how SGOL2, a member of the shugoshin family, functions in cancer than SGOL1, which has been repeatedly proved to be related to the tumorigenesis and development of cancers [20, 48–55]. It was reported that elevated expression of SGOL2 related to the abundance of tumor-infiltrating mast cells (TIMCs) indicated a poor prognosis in adrenocortical carcinoma [56]. In the previous study [57], the authors found that SGOL2 is overexpressed in HCC than adjacent tissues and related to the overall survival (OS) rate. However, they did not demonstrate that SGOL2 is an independent risk marker in HCC, which was proved in our manuscript by the prediction model based on lasso regression analysis. In another study [58], the researcher found that SGOL2 can regulate cell cycle process and bind with MAD2 in *mus musculus*, not human, not HCC. In our study, we first found the SGOL2 maybe a novel diagnostic marker in HCC, then we predicted that SGOL2 may exert its function through MAD2, which was demonstrated by vitro and vivo experiments. Furthermore, from our bioinformatic analysis part, we can also observe that the hub genes related with both SGOL2 and MAD2 are not only enriched in cell cycle related pathway, but also other pathways such as classic p53 pathway, FOXO pathway. In this study, SGOL2 mainly exerted its tumor-promoting effects by regulating MAD2 and then dysregulating the cell cycle in HCC. We first proved that SGOL2 regulates MAD2 in HCC cells, which indicates that it may serve as a potential target for molecular-based therapy. Nevertheless, there are several limitations to this study. First, further assays in vivo are required to validate the results in vitro. Second, we should also pay attention to the alterations of other pathways regulated by SGOL2 in addition to manipulating the cell cycle. Third, SK-HEP-1 cell line has been identified as a human cell line of endothelial origin [59], it is therefore not a good representation although its high expression of SGOL2.

Dysregulated transcription factors play a key role in various types of cancers [60]. To date, novel drugs targeting dysregulated transcription factors have been tested preclinically or even clinically [61]. Thus, potential proto-oncogenic transcription factors involved in the SGOL2-MAD2 pathway need further study. Through searching the JASPAR database, we found that ZNF148, PPARG::RXRA, and ETV6 bind not only SGOL2 but also MAD2 (Supplementary data). We also reported the binding sequences of ZNF-148 and ETV6 to the promoter region of SGOL2-MAD2 (Supplementary data). Moreover, ZNF-148 or ETV6 has a close correlation with SGOL2-MAD2, whereas PPARG::RXRA does not (Supplementary data). Zinc Finger Protein 148 (ZNF148), also known as ZBP-89, is a member of the Kruppel family [62]. ZNF148 could promote cell apoptosis in HCC in a p53-dependent manner or not [62–65]. It was also reported that ZNF148 is a novel tumor suppressor and a potential prognostic biomarker in HCC [66]. Moreover, the negative regulation of ZNF148 on stemness in HCC indicates its potential as a novel target to reverse cancer stem cell (CSC)-induced drug resistance [67]. E-Twenty six variant gene 6 (ETV6) belongs to the transcription factor ETS family and is associated with tumorigenesis [68]. It was reported that ETV6 promoted cell migration and invasion by directly binding to miR-429 to regulate CRKL expression in HCC [69]. Thus, both ZNF-148 and ETV6 are potential targets in HCC. In summary, our findings suggested that the functions of the SGOL2-MAD2 complex in HCC may be regulated by ZNF-148 and ETV6. However, the question of whether SGOL2-MAD2 is regulated by ZNF-148 or ETV6 requires further study.

## Conclusions

Taken together, these results suggest that SGOL2 is a novel functional oncogene in HCC and that it accelerates tumor growth via the regulation of MAD2. Moreover, SGOL2 is a potential target and clinical marker for HCC therapy. The role of SGOL2 in promoting tumorigenesis in HCC is reported here for the first time, indicating that a novel therapeutic strategy for HCC involving SGOL2 is worthy of further investigation.

## Supplementary Information

**Additional file 1.** Supplementary methods.

**Additional file 2: Fig. S1.** High expression of SGOL2 in HCC in public database. **Fig. S2.** The mRNA and protein levels of SGOL1 after the knockdown of SGOL2 in HCC cell lines. **Fig. S3.** Subgroup expression analysis of SGOL2 in HCC. **Fig. S4.** Elevated expression of SGOL2 indicated a poor prognosis in HCC patients. **Fig. S5.** SGOL2 mutations and the associations between SGOL2 and immune cells in HCC. **Fig. S6.** Genes related to SGOL2 or MAD2 in HCC. **Fig. S7.** Hub gene analysis. **Fig. S8.** The predicted transcription factors binding to SGOL2 or MAD2.

## Data Availability

Data related to this paper may be requested from the corresponding author.

## Abbreviations

HCC: hepatocellular carcinoma
CCK-8: Cell Counting Kit-8
TUNEL: Terminal deoxynucleotidyl transferase-medimed dUTP nick-endlabeling
qRT-PCR: Quantitative real-time reverse transcription PCR
IHC: Immunohistochemical
AUC: Area under the curve
ROC: Receiver operating characteristic
