# Supplementary material for: SGOL2 is a novel prognostic marker and fosters disease progression via a MAD2-mediated pathway in hepatocellular carcinoma
Source: Biomark Res. 2022 Nov 15;10:82. doi: 10.1186/s40364-022-00422-z (PMC9664666; doi:10.1186/s40364-022-00422-z)
Supplement: Supplementary file 1 — Additional file 1. Supplementary methods. [file 40364_2022_422_MOESM1_ESM.pdf]

Methods：

Mutation and immune infiltration analysis

We assessed SGOL2 mutation types and frequencies in liver cancer by the cBioPortal database and the Catalog of Somatic Mutations in Cancer (COSMIC) database. The correlation between SGOL2 expression and immune cells was measured by the Tumor Immune Estimation Resource (TIMER) database.

Protein-Protein Interaction Networks Functional Enrichment Analysis

We used the LinkedOmics database to search for differentially expressed genes related to SGOL2 and MAD2 and analyzed the RNA-Seq results in the TCGA-LIHC dataset. Then, the correlation coefficients of differentially expressed genes were determined by Spearman's test. We further used the STRING database (interaction score>0.8) and Cytoscape software (3.7.1) to construct the PPI network. We also verified the association between SGOL2 and MAD2 using the GEPIA database.

Biological function and pathway enrichment analysis

We further analyzed the clusters of the network. Gene Ontology (GO) and Kyoto Encyclopedia of Genes and Genomes (KEGG) pathway analyses were then performed via the Database for Annotation, Visualization and Integrated Discovery (DAVID) and a bioinformatics online tool to determine the enriched biological processes and KEGG pathways for subsequent analysis.

Materials：

Table 1: Antibodies for WB/Co-IP or IF for SGOL2 and MAD2

Name	WB/Co-IP	IF
SGOL2 antibody	Bethyl, A301–262A	Abcam, ab122258
MAD2 antibody	Bethyl, A300-301A	Santa Cruz, C-10
