# Supplementary material for: SGOL2 is a novel prognostic marker and fosters disease progression via a MAD2-mediated pathway in hepatocellular carcinoma
Source: Biomark Res. 2022 Nov 15;10:82. doi: 10.1186/s40364-022-00422-z (PMC9664666; doi:10.1186/s40364-022-00422-z)
Supplement: Supplementary file 2 — Additional file 2: Fig. S1. High expression of SGOL2 in HCC in public database. Fig. S2. The mRNA and protein levels of SGOL1 after the knockdown of SGOL2 in HCC cell lines. Fig. S3. Subgroup expression analysis of SGOL2 in HCC. Fig. S4. Elevated expression of SGOL2 indicated a poor prognosis in HCC patients. Fig. S5. SGOL2 mutations and the associations between SGOL2 and immune cells in HCC. Fig. S6. Genes related to SGOL2 or MAD2 in HCC. Fig. S7. Hub gene analysis. Fig. S8. The predicted transcription factors binding to SGOL2 or MAD2. [file 40364_2022_422_MOESM2_ESM.pdf]

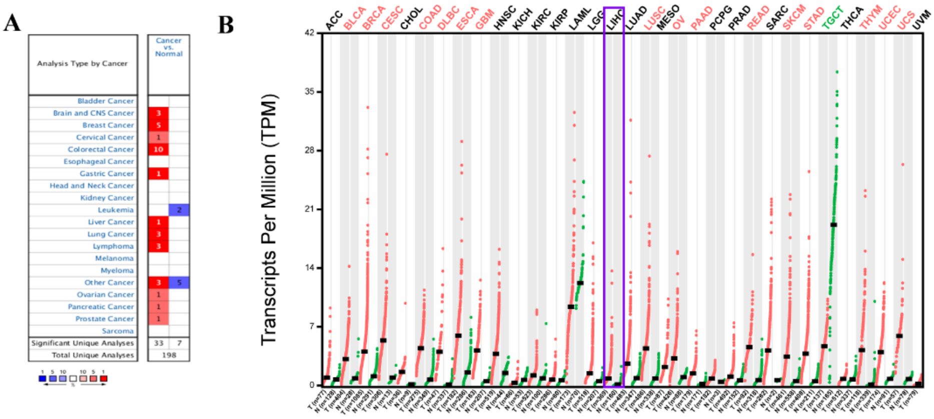

FIGURE S1 High expression of SGOL2 in HCC in public database. A, SGOL2 overexpression was detected in various types of cancers in Oncomine database. B, SGOL2 overexpression was detected in various types of cancers in GEPIA database.

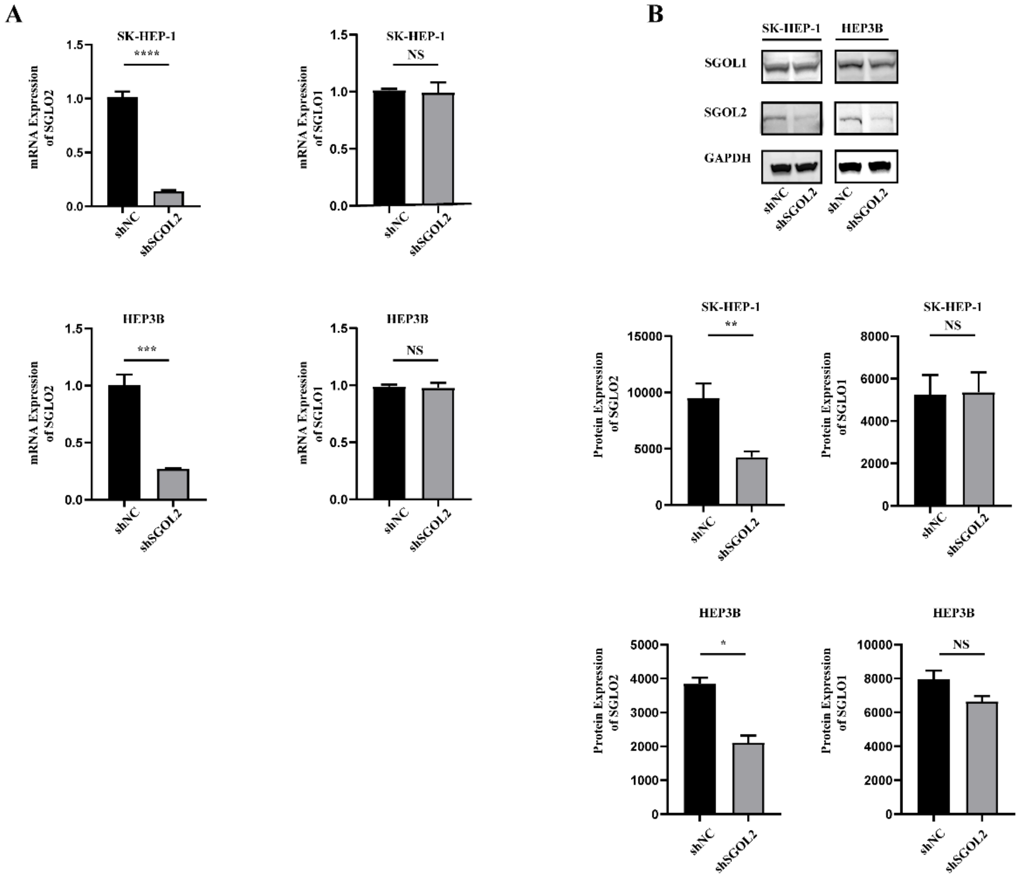

FIGURE S2 The mRNA and protein levels of SGOL1 after the knockdown of SGOL2 in HCC cell lines. A, RT–PCR. B, Western blot. *P<0.05, **P<0.01, ***P<0.001, ****P<0.0001. NS=Not statistically significant.

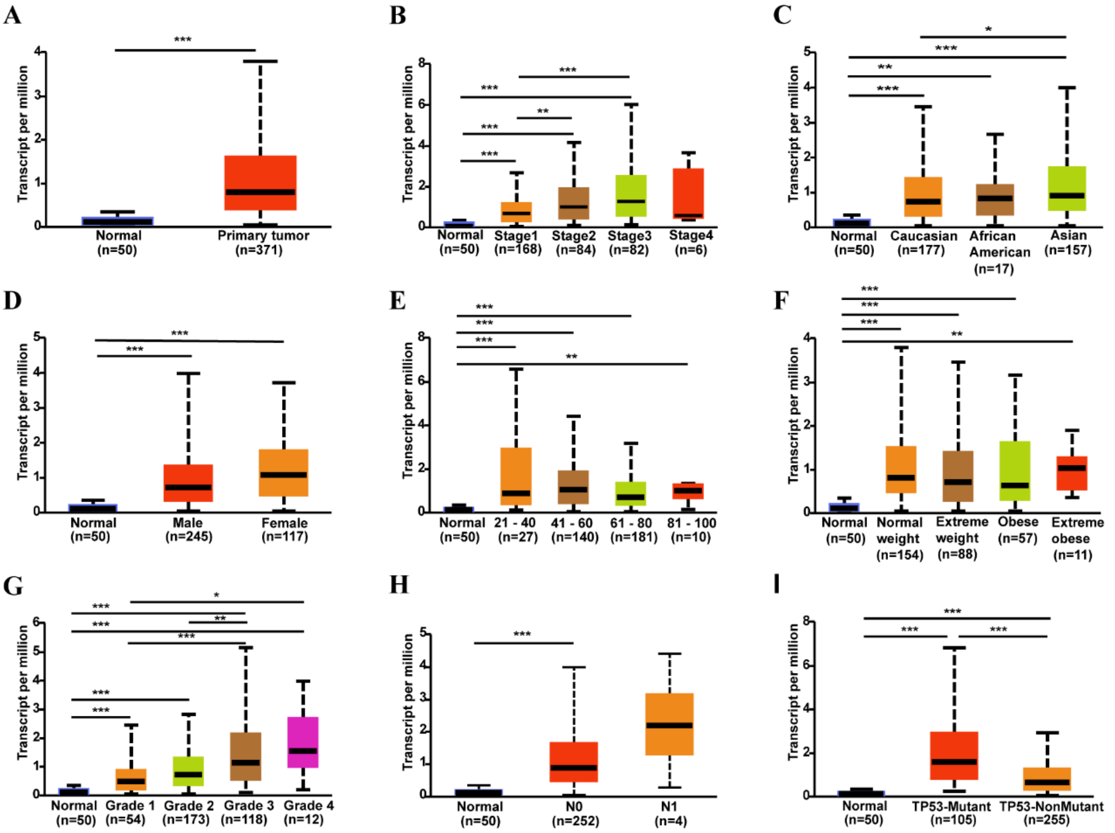

FIGURE S3 Subgroup expression analysis of SGOL2 in HCC. A, SGOL2 mRNA expression levels in controls and HCC patients. B-I, SGOL2 mRNA expression levels of HCC patients in subgroups with various races, genders, ages, weights, tumor grades, metastatic statuses, and with TP-53 mutation or no TP-53 mutation. *P<0.05, **P<0.01, ***P<0.001, ****P<0.0001.

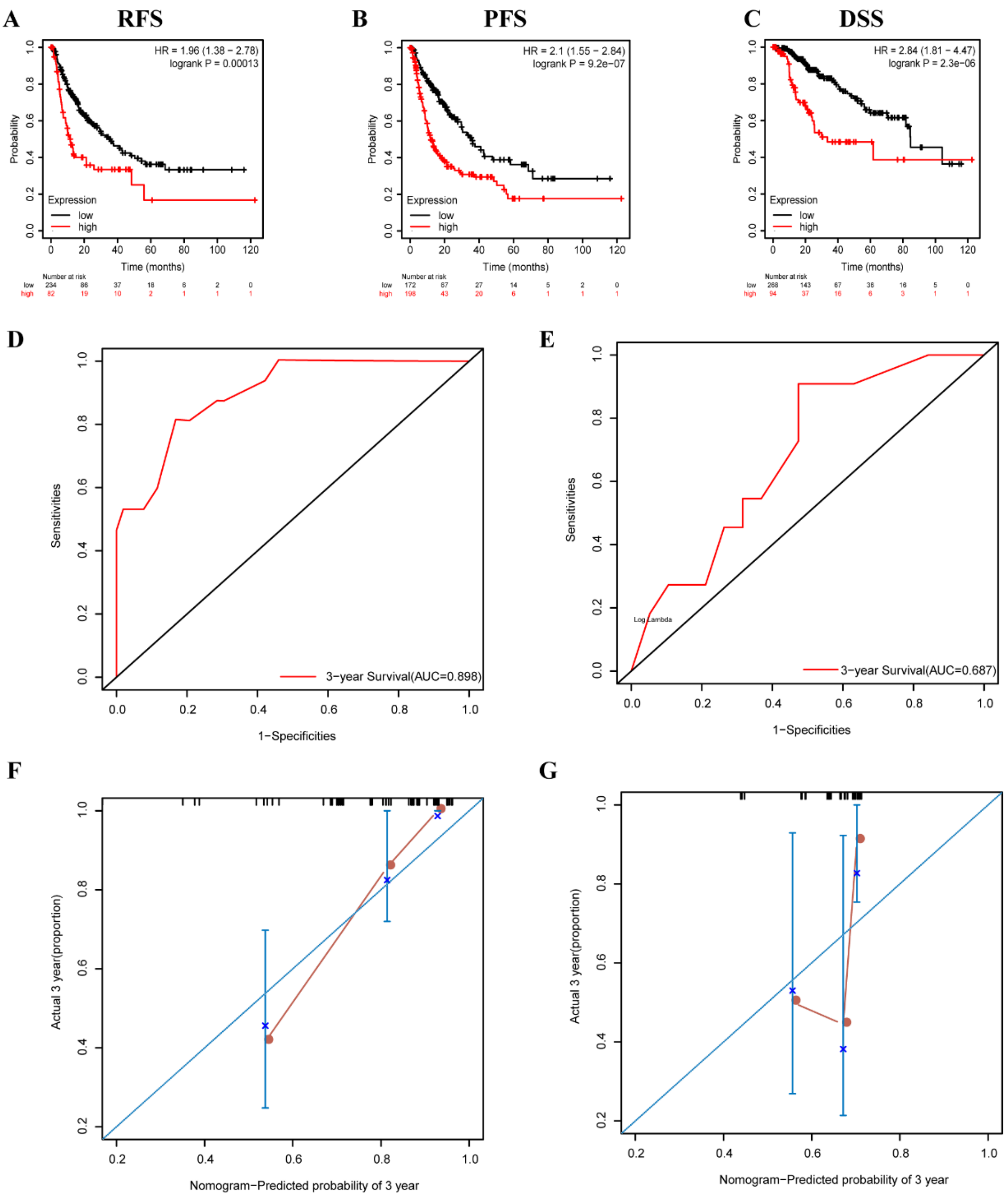

FIGURE S4 Elevated expression of SGOL2 indicated a poor prognosis in HCC patients. A-C, The prognostic value of SGOL2 in HCC patients based on the Kaplan–Meier Plotter database. D-E, ROC curve analysis was used to evaluate the performance of this nomogram for 3-year overall survival prediction in the training and validation groups. L-M, 3-year calibration curve in the training (left) and validation groups (right). OS, overall survival; RFS, relapse-free survival; PFS, progression-free survival; DSS, disease-specific survival; HR, hazard ratio.

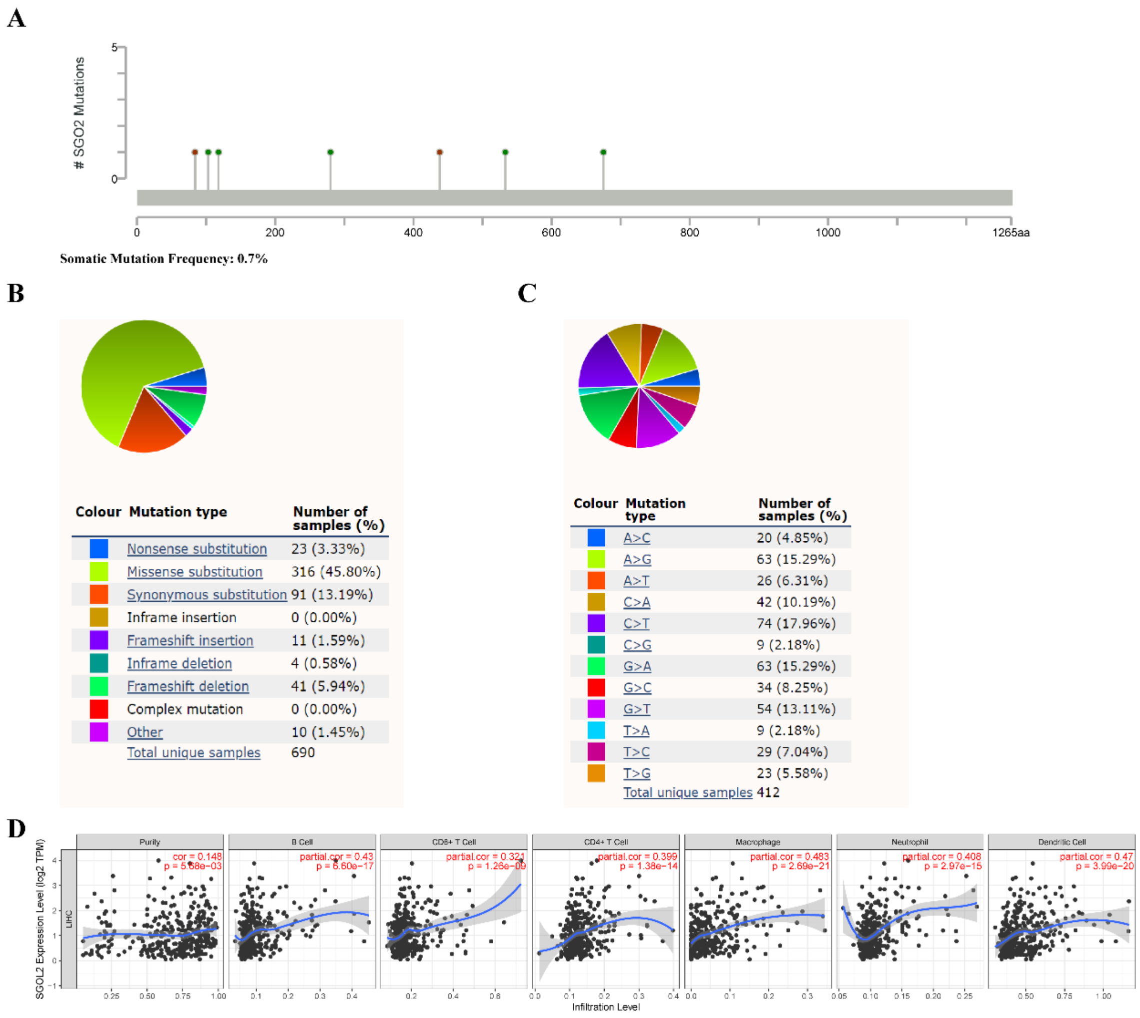

FIGURE S5 SGOL2 mutations and the associations between SGOL2 and immune cells in HCC. A-C, The representation of SGOL2 mutations in HCC (cBioPorta and COSMIC); D, SGOL2 is associated with immune infiltration in HCC (TIMER).

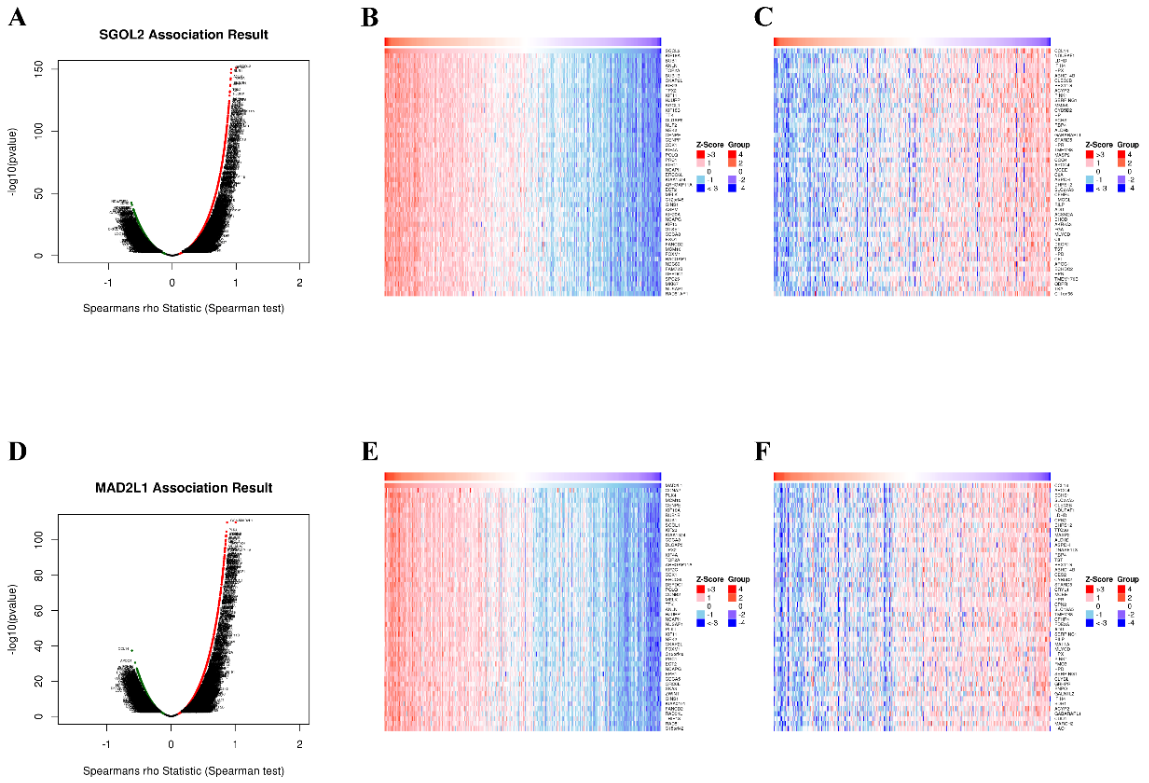

FIGURE S6 Genes related to SGOL2 or MAD2 in HCC. A, Differentially expressed genes related to SGOL2. B-C, Genes positively or negatively related to SGOL2 (top 50). D, Differentially expressed genes related to MAD2. E-F, Genes positively or negatively related to MAD2 (top 50).

A

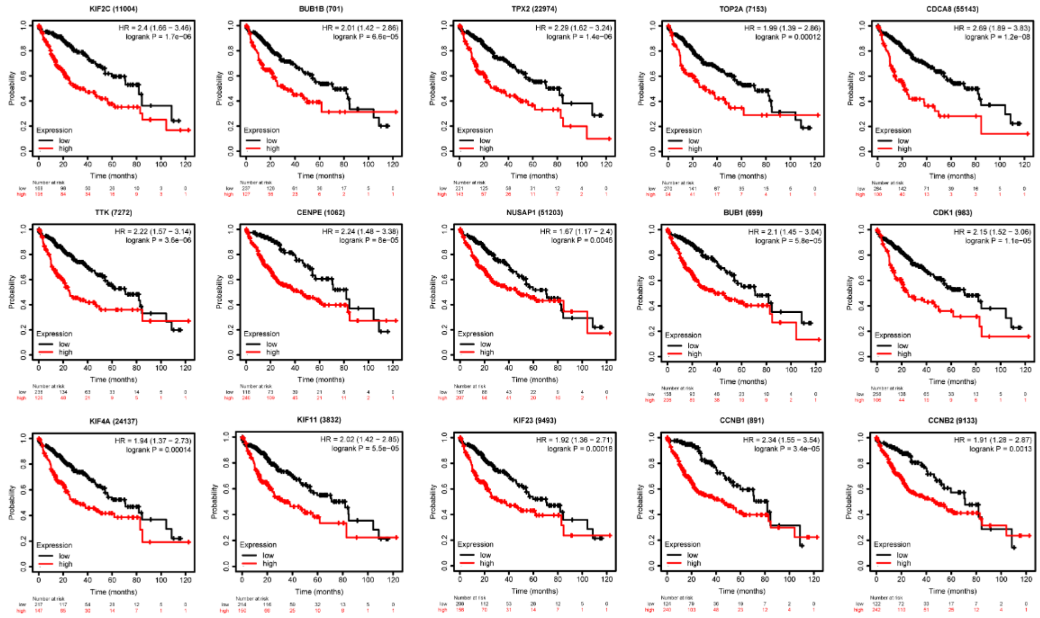

FIGURE S7 Hub gene analysis. A, The prognostic values of the top 15 hub genes.

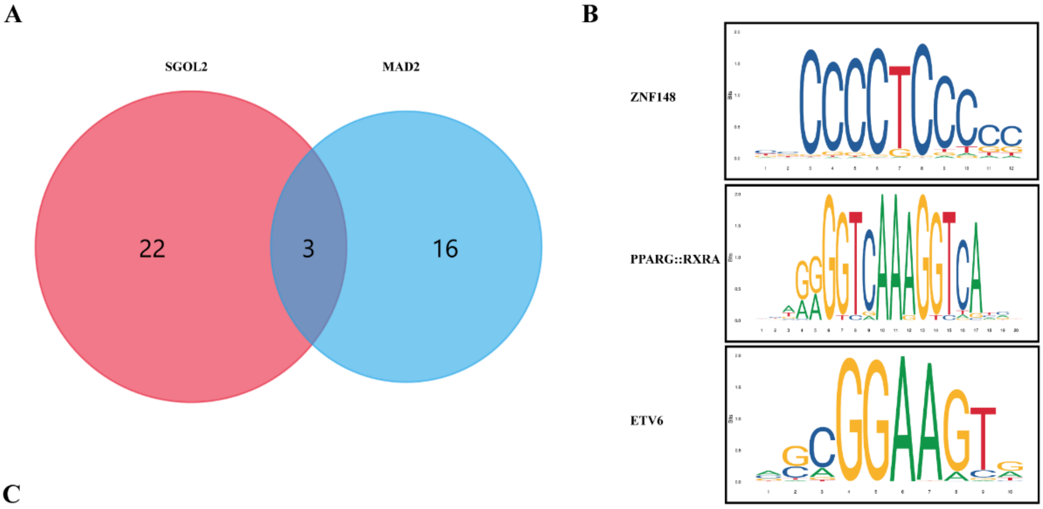

C

Matrix ID	Name	Score	Relative score	Sequence ID	Start	End	Strand	Predicted sequence
MA0645.1	ETV6	15.3643	0.991109457	NC_000002.12:200508198-200510297	1863	1872	+	CGCGGAAGTG
MA1653.1	ZNF148	15.3179	0.938892238	NC_000002.12:200508198-200510297	2001	2012	+	CGCCCCGCCCCC
MA1653.1	ZNF148	12.6147	0.89313652	NC_000002.12:200508198-200510297	1779	1790	-	CGCCCCACCCCC

D

Matrix ID	Name	Score	Relative score	Sequence ID	Start	End	Strand	Predicted sequence
MA0645.1	ETV6	14.3489	0.973719065	NC_000004.12:c120068848-120066749	1623	1632	+	AGCGGAAGTT
MA0645.1	ETV6	13.1497	0.953181407	NC_000004.12:c120068848-120066749	120	129	-	AGAGGAAGTA
MA0645.1	ETV6	11.4355	0.923824987	NC_000004.12:c120068848-120066749	2039	2048	+	CGGGGAAGTG
MA1653.1	ZNF148	14.0646	0.917678524	NC_000004.12:c120068848-120066749	644	655	+	ATCCCCTCCCCT

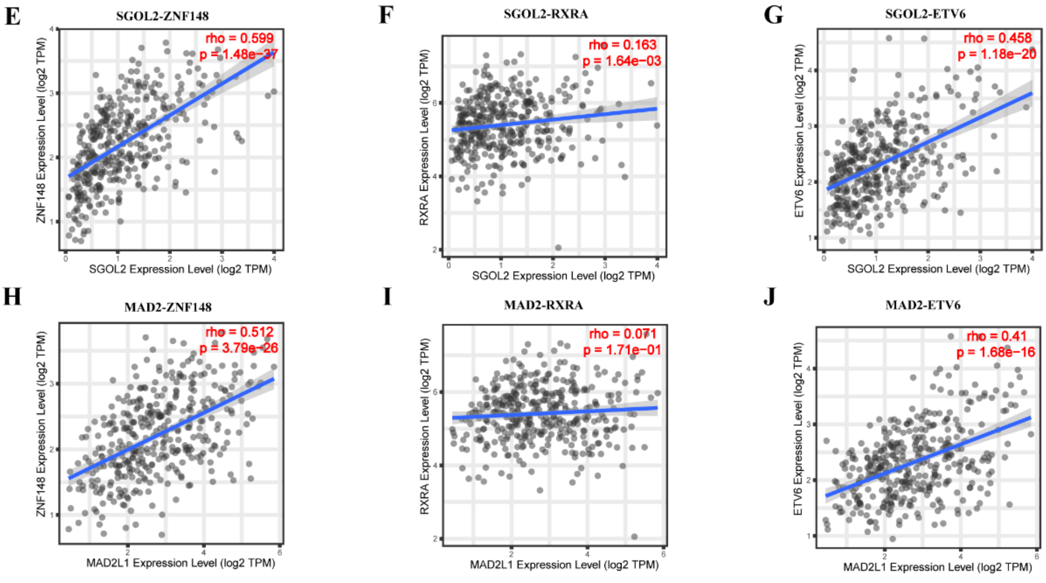

FIGURE S8 The predicted transcription factors binding to SGOL2 or MAD2. A, The predicted transcription factors binding to SGOL2 or MAD2 were visualized in a Venn diagram using the JASPAR database. B, The sequences of the three transcription factors. C-D, The transcription factor-binding site of ZNF148 or ETV6 in the gene promoter of SGOL2 or MAD2. E-J, ZNF148, or ETV6 is positively correlated with

SGOL2 or MAD2 in HCC (TIMER).
